# Do natural or synthetic excito-repellents work better? A study on coastal malaria vector *Anopheles epiroticus* in Ko Chang, Thailand

**DOI:** 10.7717/peerj.21237

**Published:** 2026-05-15

**Authors:** Chutipong Sukkanon, Supinya Jabjai, Wanapa Ritthison, Theeraphap Chareonviriyaphap, Jirod Nararak

**Affiliations:** 1 Department of Clinical Microscopy, Faculty of Medical Technology, Mahidol University, Nakhon Pathom, Thailand; 2 Office of Disease Prevention and Control, Region 6 Chonburi, Ministry of Public Health, Chonburi, Thailand; 3 Department of Entomology, Faculty of Agriculture, Kasetsart University, Bangkok, Thailand; 4 Royal Society of Thailand, Bangkok, Thailand; 5 Research and Lifelong Learning Center for Urban and Environmental Entomology, Kasetsart University Institute for Advanced Studies, Kasetsart University, Bangkok, Thailand

**Keywords:** *Anopheles epiroticus*, Excito-repellency, Malaria, Mosquito control, *Plasmodium knowlesi*

## Abstract

Malaria remains a significant public health concern in Ko Chang, an island in Trat Province, eastern Thailand. The island has historically experienced low to moderate malaria transmission, particularly in forested and coastal zones. Transmission is driven by local *Anopheles* vectors such as *Anopheles epiroticus* Linton & Harbach. In this study, we employed the excito-repellency (ER) assay system to evaluate behavioral responses of laboratory and field strains of *An. epiroticus* to four synthetic and two natural repellent agents. Mosquito escape responses (% escape) were recorded under contact and non-contact conditions to determine irritant and repellent properties. For the laboratory strain, deltamethrin (42%) and permethrin (35%) at LC_50_ levels induced the highest escape responses in contact trials. In contrast, alpha-cypermethrin (4–14%) induced limited irritancy and repellency. Citronella oil (>50%) at 5.0% (v/v) elicited the strongest non-contact repellency, outperforming DEET (18–26%) and vetiver oil (26–40%). However, the field strain showed a different pattern: DEET (64%) and deltamethrin (60%) were most effective in contact trials, while citronella and vetiver oils (both ∼14%) had minimal non-contact effects. Our findings indicate differences in excito-repellency between field and laboratory strains of *An. epiroticus* in Ko Chang. Deltamethrin, permethrin, and DEET were more effective as contact irritants against the field population, whereas citronella oil demonstrated greater efficacy as non-contact repellents against the laboratory strain. These results indicate the need to account for behavioral variation when designing vector control strategies and inform both malaria prevention for travelers and local control programs in coastal Thailand.

## Introduction

Malaria remains one of the most pressing public health challenges in tropical and subtropical regions. Southeast Asia bears a significant burden due to its environmental diversity and the coexistence of multiple vector species that sustain complex transmission cycles (Tananchai et al., 2019). In recent years, *Plasmodium knowlesi* has emerged as an important contributor to malaria cases in South-East Asia, including Thailand (Ahebwa et al., 2025). More than 3,000 cases of *P. knowlesi* infection were reported globally in 2023, an 18.9% increase compared with 2022. The number of indigenous cases also rose by 22% over the same period (WHO, 2024). *Plasmodium knowlesi* is 24 times more likely to cause infection than other zoonotic *Plasmodium* species (Ahebwa et al., 2025). In Thailand, reported *P. knowlesi* cases increased markedly from 72 in 2021 to 275 in 2023 (DVBD, 2021; DVBD, 2023). This growing burden and ongoing transmission pose unique challenges to malaria elimination efforts and may affect progress toward malaria-free certification.

*Anopheles epiroticus* Linton & Harbach (Diptera: Culicidae), previously classified as *Anopheles sundaicus*, plays a pivotal role in the malaria epidemiology of Thailand’s coastal and brackish water areas (Overgaard, Suwonkerd & Hii, 2015). *Anopheles epiroticus* thrives in saline and semi-saline habitats, often associated with mangroves and aquaculture zones (Chaiphongpachara & Laojun, 2020). Behavioral plasticity of *An. epiroticus*, particularly its feeding and resting preferences, can reduce the effectiveness of conventional indoor residual spraying (IRS) and long-lasting insecticidal nets (LLINs) (Ritthison et al., 2014a).

Insecticides and repellents employed for mosquito control are the most common. Pyrethroids, particularly deltamethrin and permethrin, are widely used for targeting adult mosquitoes, as endorsed by the World Health Organization (WHO, 2022). In Thailand, the Ministry of Public Health has used these pyrethroid insecticides for space spraying with fogging or misting machines, as well as for mosquito net treatments. Both deltamethrin and permethrin trigger strong contact irritant responses in mosquitoes (Chareonviriyaphap et al., 2013; Kongmee et al., 2004). Topical and spatial repellent use has increased in recent years, especially in response to outdoor malaria and arboviral transmission. These often occur during times and in locations where traditional insecticide-based interventions (*e.g.*, indoor residual spraying and insecticide-treated nets) are less effective or practical. Many vector species exhibit exophagic and exophilic behaviors and avoid treated surfaces (John Ravindran & Eapen, 2024). This indicates a need to evaluate and improve repellent-based personal protection tools that can be applied directly to the skin or used in the surrounding environment (Luker, 2024).

Behavioral responses in insects triggered by insecticides are generally categorized into irritation and repellency. Irritation refers to the reaction where an insect departs from a treated surface following physical tarsal contact with the insecticide. In contrast, spatial repellency—or avoidance—describes the capacity of a chemical to provoke an avoidance response, compelling insects to move away from the stimulus without direct contact, thus reducing the likelihood of interaction with the treated surface. The behavioral responses of irritancy and repellency are important considerations in the design and evaluation of mosquito control interventions. These behaviors can be quantitatively assessed using an excito-repellency test system (ER), that measures mosquito responses to insecticide-treated surfaces under controlled conditions (Roberts et al., 1997). The Excito-Repellency (ER) assay technique is used to assess the sublethal effects of synthetic or naturally derived chemical compounds such as contact excitation and non-contact repellency properties (Bhoopong, Chareonviriyaphap & Sukkanon, 2022).

This study evaluates the efficacy of selected synthetic (deltamethrin, permethrin, alpha-cypermethrin, and DEET) and natural (citronella and vetiver oils) excito-repellent agents against *An. epiroticus* in Ko Chang, Thailand. By comparing responses of laboratory and field populations under controlled experimental conditions, the research aims to elucidate differential behavioral patterns and inform the optimization of vector control strategies for coastal malaria-endemic regions. This comprehensive assessment contributes to the understanding of the relative effectiveness of synthetic and natural repellents, with implications for sustainable malaria vector management.

## Materials & Methods

### *Anopheles epiroticus* Linton & Harbach

An insecticide-susceptible laboratory strain of *An. epiroticus* s.s. (SGP strain) was used in this study (Phoomkhong, Bangs & Chareonviriyaphap, 2018). This colony has been maintained at the Department of Entomology, Faculty of Agriculture, Kasetsart University (KU), Bangkok, since 2015, and that it has originated from Novartis (Singapore) Pte Ltd. (SGP). Larvae were reared (Phoomkhong, Bangs & Chareonviriyaphap, 2018) inbrackish water (0.6–0.7% NaCl solution) at 25 ± 2 °C, 80 ± 10% relative humidity with a 12:12 h cycle of light:dark phase. Larval food (TetraMin^®^ Tropical Fish Food Flakes, Tetra, Virginia, USA) was provided three times daily. Surface films were removed daily. Water changed every 2–3 days. Pupae were collected daily in small cups and placed in wire-mesh cages (30 × 30 × 30 cm) for adult emergence. Adults were allowed to feed on 10% sucrose solution *ad libitum*. Pathogen-negative human-donated blood obtained from Thai Red Cross was provided using an artificial membrane feeding system (Phasomkusolsil et al., 2013) for egg production.

**Figure 1:**
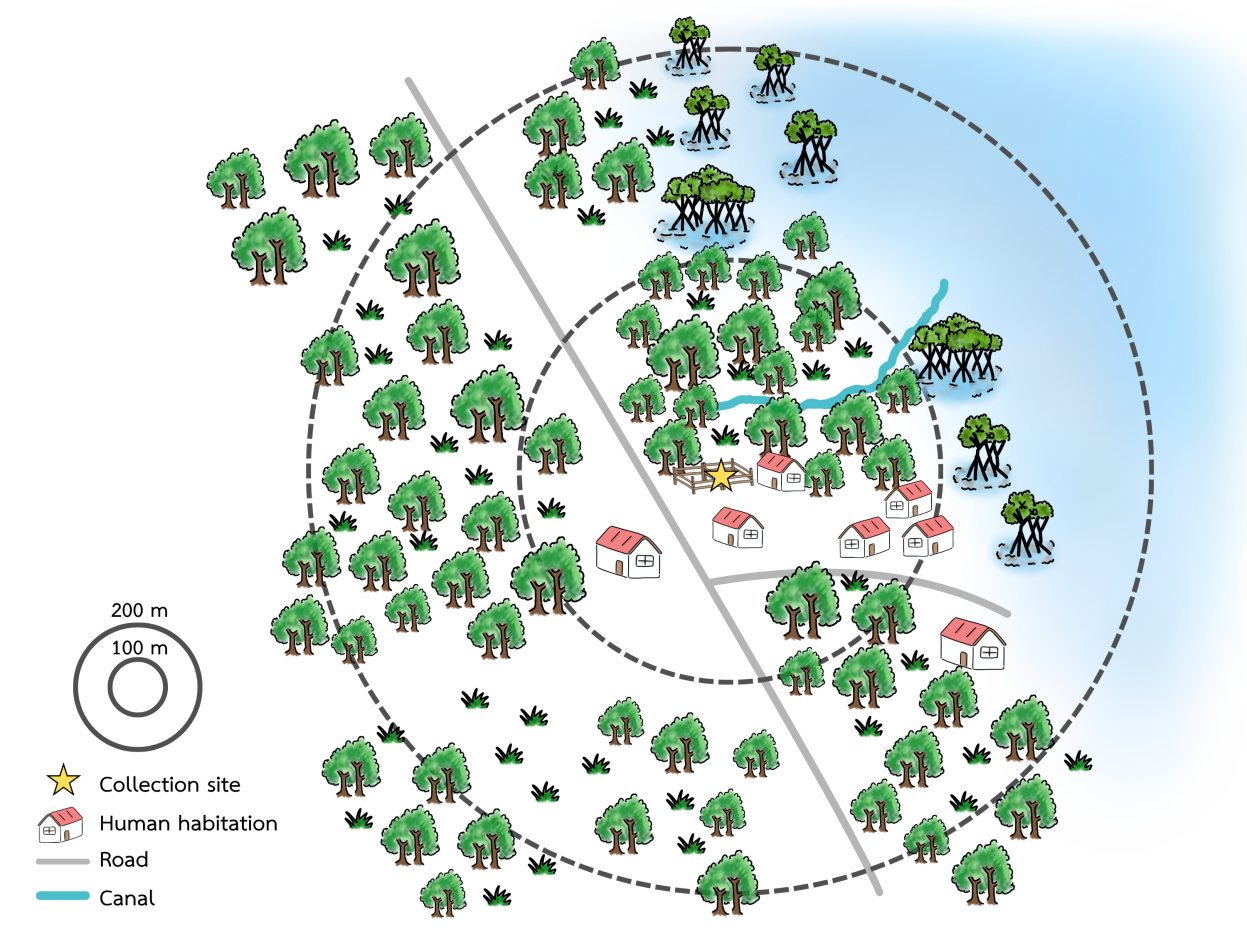
Illustration of field collection sites and surrounding area. A coastal bay landscape with pond margins and a freshwater stream that connects to the sea (light blue area on the right)—the ecology is highly suitable for *An. epiroticus*, a mosquito strongly associated with coastal lowlands and water bodies influenced by salinity gradients. The stream–estuary interface creates fresh-to-brackish transitional habitats that shift with tides and seasonal rainfall, while nearby pond edges, drainage channels, and stagnant sections can provide stable, sunlit larval habitats with organic matter and algal growth, conditions commonly reported for *An. epiroticus* breeding (including use of fresh, brackish, and even saline water in coastal Thailand). Created using Canva (https://www.canva.com/).

The field strain of *An. epiroticus* s.l. was derived from fed females collected using and entomological mount aspirator around the buffalo pen between 18:00 h to 21:00 h. All buffaloes had already been maintained within the collection area of the Koh Chang Shooting Range (12°03′47.5″N 102°22′51.2″E), Ko Chang District, Trat Province, eastern Thailand, near the Cambodian border. The collection site is located close to the sea (<200 m), approximately 39 m above sea level, and is surrounded by fruit orchards and rubber plantations, along with native mangroves. The nearest human habitation areas were within a 100 m radius (Fig. 1). During the mosquito collection process, all buffaloes were under the care of their owner. Most female *An. epiroticus* s.l. were collected from fences surrounding the buffalo pens not directly from the buffaloes. Mosquitoes were placed in a holding cup, identified using morphological keys (Rattanarithikul et al., 2006), and transferred back to KU insectary for further rearing. Gravid females were provided a small cup containing artificial brackish water inside wire-mesh cages for oviposition. Hatched larvae were reared as previously described. Only F_1_ generation was used in the study. Due to the limited number of F_1_ generation mosquitoes, susceptibility testing of the field strain to pyrethroids was not performed. This protocol was approved by Kasetsart University’s Institutional Animal Care and Use Committee (Reference No. ACKU68-ETC-009).

### Excito-repellent agents and treated paper

A total of four synthetic and two natural repellent agents were used in the present study. The three pyrethroids were: (1) deltamethrin (98% AI; Lot no. DCM21512293), provided by BASF Thailand; (2) permethrin (92.15% AI), and (3) alpha-cypermethrin (97.34% AI), Sherwood Chemicals Public Company Limited, Suan Luang, Bangkok. DEET (*N*, *N*-diethyl-meta-toluamide; Lot. no: MKBH0428V) 97% AI (Sigma-Aldrich^®^, St. Louis, MO, USA) was used as a standard. Vetiver oil (Lot. no: 25090698-1) and citronella oil (*Cymbopogon nardus* (L.) Rendle essential oil (Lot. no: MK-40012) were purchased from Thai-China Flavours and Fragrances Industry Co., Ltd. (Ayutthaya, Thailand).

Deltamethrin, permethrin, and alpha-cypermethrin were dissolved in mixture of acetone (Baker Analyzed™ A.C.S. reagent, J.T. Baker™, Fisher Scientific International, Inc., USA) and silicone oil (Dow Corning^®^ 556 silicon oil, Dow Chemical Company and Corning, Inc., MI, USA) to obtain previously established LC_50_ and diagnostic concentrations (DC) for *An. epiroticus* (Phoomkhong, Bangs & Chareonviriyaphap, 2018). The LC_50_ of deltamethrin, permethrin, and alpha-cypermethrin are 0.00035%, 0.01030%, and 0.00046%, respectively, while the DC are 0.006%, 0.349%, and 0.009%, respectively. DEET, vetiver oil, and citronella oil were diluted to concentrations of 2.5% and 5% in absolute ethanol (Merck, Darmstadt, Germany), concentrations that as optimal for mosquito-repellent efficacy (Nararak et al., 2016; Sathantriphop et al., 2014). Whatman No. 1 filter papers (14.7 × 17.5 cm) were impregnated with 2.8 mL of test solution and solvent applied to control papers. Impregnated papers air dried on aluminum foil at room temperature for 60 min before use. Papers were prepared and used only once.

### Mosquito behavioral assay

The excito-repellent property of the selected synthetic and natural-based compounds against *An. epiroticus* was evaluated using the ‘excito-repellency (ER) assay system’ originally developed by Roberts et al. (1997) and improved by Chareonviriyaphap, Prabaripai & Sungvornyothrin (2002). The schematic design of the ER assay system has been previously published (Bhoopong, Chareonviriyaphap & Sukkanon, 2022; Boonyuan et al., 2024). Full configuration of the ER system consists of four chambers: (1) non-contact control and treatment chambers; and (2) contact control and treatment chambers. There are two types of chambers in the ER system. First, to test repellency, treated papers were placed behind a mesh screen barrier in the chambers, called the ‘non-contact chamber.’ In this setup, mosquitoes were only exposed to airborne compound molecules, preventing direct tarsal contact with the treated papers. Second, in the ‘contact chamber,’ irritancy is measured by allowing mosquitoes direct physical contact with the treated paper. Compound exhibiting ER cause the exposed mosquito to display an avoidance behavior by moving away from the compound source (*i.e.,* treated papers). The mosquito escapes from the treatment chamber into a connected, compound-free box, where the experimenter observes and counts them. Hence, the escape response is the main outcome, with a high escape percentage indicating strong ER properties. Additionally, knockdown and mortality can also be recorded after 30 min of exposure time.

Twelve hours prior to the experiment sugar meal was replaced with water in the cage of 4- to 8-day-old mosquitoes. The experimental room was set at 25 ± 2 °C and 80 ± 10% relative humidity. All tests were performed between 08:00 to 16:30 h. For each replication, a mouth aspirator was used to introduce 15 active females into each of the four chambers. The test mosquitoes were then given a 3-minute acclimation period after the chamber was closed. The exit portal slot of each chamber was opened to start the testing. The number of escaped mosquitoes was counted at 1-minute intervals over a 30-minute period. Escaped mosquitoes were removed from the receiving chamber and transferred to holding cups containing a sugar meal. The non-escaped mosquitoes were later removed from each of the four chambers and placed in separate holding cups after 30 min of exposure time. The number of knockdowns was immediately recorded. Both escaped and non-escaped mosquitoes were held under rearing conditions for 24 h for mortality observation. Then a new cohort of 15 females per ER chamber was used for the new replication (60 mosquitoes/replica). Four replications were performed. A total of 240 females were used for each of the test compounds. All behavioral assays on the F_1_ generation of the field population of *An. epiroticus* s.l. were performed under laboratory conditions.

### Statistical analysis

GraphPad Prism (GraphPad Software, San Diego, CA, USA) was used for all data recording, editing, and entry. Final escape proportions (%) and standard errors of the mean (SEM) were calculated from replicate assays. The mean percentages of knockdown and mortality were calculated separately for the escaped and non-escaped groups, using the total number of mosquitoes within each respective group as the denominator, rather than the total number of mosquitoes per replicate. The Mann–Whitney U test was applied to compare final escape proportions between non-contact and contact trials at the same concentrations. The Kruskal–Wallis test followed by Dunn’s Post Hoc Multiple Comparison test was applied to compare final escape proportions among test compounds at the same concentration. Statistical significance was set at *P* < 0.05. Abbott’s formula (Abbott, 1925) was applied to adjust for final escape proportions if the outcome in matched control chambers ranged between 5% and 20%. Control escape rates were monitored to ensure they remained within the acceptable range; therefore, no separate statistical comparison of control escape rates was performed. To evaluate the mosquito behavioral response (termed ‘escape pattern’), Kaplan–Meier survival analysis was applied to the initial escape data recorded at 1-minute intervals. Non-escaped and escaped mosquitoes were treated as ‘survived’ and ‘event’, respectively, for computational purposes (Roberts et al., 1997). A log-rank method (Mantel & Haenszel, 1959) was then applied to compare differences in escape patterns between control and treatment chambers, non-contact and contact trials, laboratory strain and field population, and between compound concentrations. Log-rank significance reflects differences in escape pattern (time-to-escape) and does not necessarily indicate differences in final escape proportions. Statistical significance was set at 95% confidence interval (*P* < 0.05). Log-rank tests were used to compare escape-time distributions. In addition to statistical significance, final corrected escape proportions were examined to provide context for effect size and practical relevance. The details of statistical analysis of the ER assay system have been previously described (Roberts et al., 1997).

## Results

**Figure 2:**
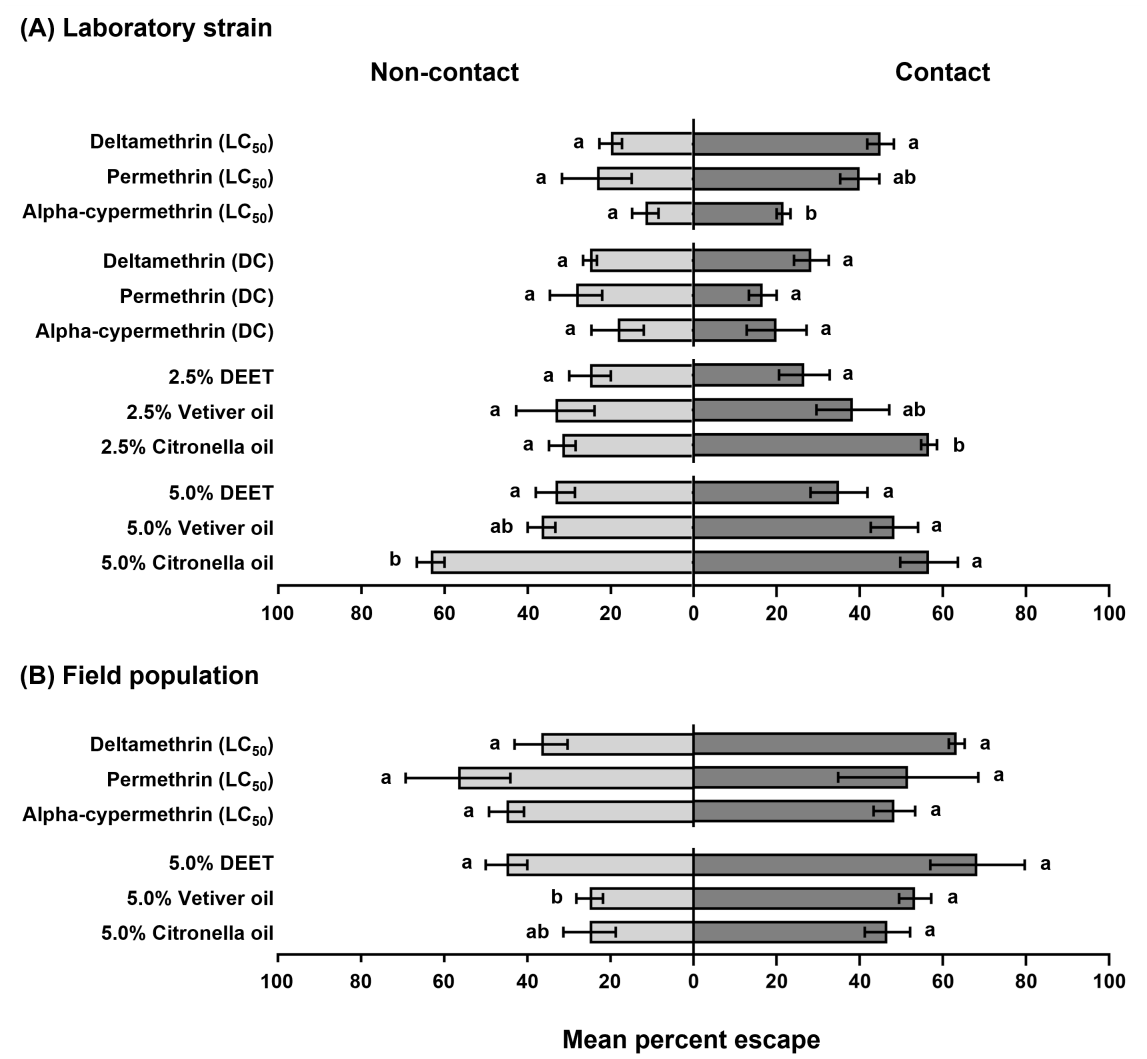
Final escape proportions (% ± SE) of *An. epiroticus* (A) laboratory strain and (B) field strain exposed to various synthetic and natural repellents using the ER assay system. Different letters beside each bar indicate significant differences (*P* < 0.05) in mean percent escape between repellents within the same treatment group (non-contact or contact) at the same concentration, as determined by Dunn’s multiple comparisons test.

The final escape proportions (%±SEM) of *An. epiroticus* exposed to four synthetic and two natural repellent agents is presented in Tables 1 and 2. Escape proportions was similar between the non-contact and contact trials in each control group, regardless of the test compound. Higher escape proportions were observed in all treatments of all test compounds compared to the respective control groups. For the *An. epiroticus* laboratory strain, all pyrethroids had similar escape proportions between the non-contact and contact trials at the same concentration, except for deltamethrin at LC_50_ (*P* = 0.0286) (Table 1S). In the contact trial, the LC_50_ of deltamethrin and permethrin elicited the highest escape proportions after adjustment using Abbott’s formula. However, for the DC, both pyrethroids had similar escape responses ranging from 12.2% to 23.2%. Lower escape proportions were observed for alpha-cypermethrin at both LC_50_ and DC in both non-contact and contact trials (Table 1). For DEET, the strongest escape responses were found in the contact (30.3%) and non-contact (25.9%) trials at the 5.0% concentration. However, no significant differences in final escape proportions were observed between the trials, regardless of DEET concentration (Table 1 and 1S). Vetiver oil at 2.5% and 5.0% concentrations yielded no significant differences in escape proportions between trials, ranging from 27.2% to 31.4% and 26.9% to 40.3%, respectively. In contrast, citronella oil at a 5.0% concentration elicited the highest adjusted escape responses in both non-contact (59.2%) and contact (52.7%) trials. The contact trial of 2.5% citronella oil caused a significantly 1.84-times higher escape response compared to the non-contact trial (*P* = 0.0286; Table 1 and 1S). When final escape proportions were compared within groups of the same concentration, almost all test compounds produced no significant differences in escape response in both non-contact and contact trials (Fig. 2). Significant differences were found in only three comparison groups. Citronella oil caused significantly more mosquitoes to escape than DEET at both 2.5% and 5.0% concentrations in the contact and non-contact trials, respectively. In the contact trial, deltamethrin also significantly elicited a higher escape response compared to alpha-cypermethrin in the LC_50_ group (Fig. 2).

**Table 1:** Final escape proportions (% ± SEM) of female *An. epiroticus* laboratory strain exposed to various synthetic and natural repellents using ER assay system.

**Repellents**	**ER assay**	**Percent escape**		
		**Control**	**Treatment**	% Corrected^*^
Deltamethrin (LC_50_)	Non-contact	8.33 ± 3.19	20.00 ± 2.72^a^	12.73
	Contact	5.00 ± 3.19	45.00 ± 3.19^b^	42.11
Deltamethrin (DC)	Non-contact	8.33 ± 1.67	25.00 ± 1.67^a^	18.18
	Contact	10.00 ± 1.92	28.33 ± 4.19^a^	20.37
Permethrin (LC_50_)	Non-contact	10.00 ± 3.33	23.33 ± 8.39^a^	14.81
	Contact	8.33 ± 1.67	40.00 ± 4.71^a^	34.55
Permethrin (DC)	Non-contact	6.67 ± 0.00	28.33 ± 6.31^a^	23.21
	Contact	5.00 ± 3.19	16.67 ± 3.33^a^	12.28
Alpha-cypermethrin (LC_50_)	Non-contact	8.33 ± 1.67	11.67 ± 3.19^a^	3.64
	Contact	13.33 ± 2.72	21.67 ± 1.67^a^	9.62
Alpha-cypermethrin (DC)	Non-contact	6.67 ± 2.72	18.33 ± 6.31^a^	12.49
	Contact	6.67 ± 2.72	20.00 ± 7.20^a^	14.28
2.5% DEET	Non-contact	8.33 ± 3.19	25.00 ± 5.00^a^	18.18
	Contact	8.33 ± 4.19	26.67 ± 6.09^a^	20.01
5.0% DEET	Non-contact	10.00 ± 3.33	33.33 ± 4.71^a^	25.92
	Contact	6.67 ± 0.00	35.00 ± 6.87^a^	30.35
2.5% Vetiver oil	Non-contact	8.33 ± 1.67	33.33 ± 9.43^a^	27.27
	Contact	10.00 ± 1.92	38.33 ± 8.77^a^	31.48
5.0% Vetiver oil	Non-contact	13.33 ± 3.85	36.67 ± 3.33^a^	26.93
	Contact	13.33 ± 0.00	48.33 ± 5.69^a^	40.38
2.5% Citronella oil	Non-contact	5.00 ± 3.19	31.67 ± 3.19^a^	28.07
	Contact	10.00 ± 1.92	56.67 ± 1.92^b^	51.86
5.0% Citronella oil	Non-contact	10.00 ± 4.30	63.33 ± 3.33^a^	59.26
	Contact	8.33 ± 1.67	56.67 ± 6.94^a^	52.73

**Notes.**

* Percent escape adjusted with paired controls using Abbott’s formula.

Different letters beside percent escape of treatment group indicate significant differences (*P* < 0.05) in mean percent escape between non-contact or contact in the same test compound of the same concentration using Mann–Whitney U test.

% w/v means percent of weight (g) of repellent in the total volume of solution. LC_50_; lethal concentration 50, DC; diagnostic concentration. LC_50_ of deltamethrin, permethrin, and alpha-cypermethrin are 0.00035%, 0.01030%, and 0.00046%, respectively. DC of deltamethrin, permethrin, and alpha-cypermethrin are 0.006%, 0.349%, and 0.009%, respectively.

**Table 2:** Final escape proportions (% ± SEM) of female *An. epiroticus* field strain exposed to various synthetic and natural repellents using ER assay system.

**Repellents**	**ER assay**	**Percent escape**		
		**Control**	**Treatment**	% Corrected^*^
Deltamethrin (LC_50_)	Non-contact	13.33 ± 4.71	36.67 ± 6.38^a^	26.93
	Contact	8.33 ± 1.67	63.33 ± 1.92^b^	60.00
Permethrin (LC_50_)	Non-contact	8.33 ± 1.67	56.67 ± 12.62^a^	52.73
	Contact	8.33 ± 1.67	51.67 ± 16.86^a^	47.28
Alpha-cypermethrin (LC_50_)	Non-contact	8.33 ± 4.19	45.00 ± 4.19^a^	40.00
	Contact	15.00 ± 3.19	48.33 ± 5.00^a^	39.21
5.0% DEET	Non-contact	11.67 ± 3.19	45.00 ± 5.00^a^	37.73
	Contact	11.67 ± 5.00	68.33 ± 11.34^a^	64.15
5.0% Vetiver oil	Non-contact	15.00 ± 1.67	25.00 ± 3.19^a^	11.76
	Contact	10.00 ± 1.92	53.33 ± 3.85^b^	48.14
5.0% Citronella oil	Non-contact	6.67 ± 2.72	20.00 ± 6.09^a^	14.28
	Contact	10.00 ± 1.92	46.67 ± 5.44^a^	40.74

**Notes.**

* Percent escape adjusted with paired controls using Abbott’s formula.

Different letters beside percent escape of treatment group indicate significant differences (*P* < 0.05) in mean percent escape between non-contact or contact in the same test compound of the same concentration using Mann–Whitney U test.

% w/v means percent of weight (g) of repellent in the total volume of solution. LC_50_; lethal concentration 50. LC_50_ of deltamethrin, permethrin, and alpha-cypermethrin are 0.00035%, 0.01030%, and 0.00046%, respectively.

For the field population, relatively more escape proportions were observed in the contact trial compared to the non-contact trials, except for permethrin and alpha-cypermethrin (Table 2). The established LC_50_ value of deltamethrin produced a significantly stronger escape proportion (60%) in the contact trial (*P* = 0.0286) while escape responses for permethrin and alpha-cypermethrin were similar between trials (Table 2 and 2S). For 5.0% DEET, the highest escape proportion was also observed in the contact trial (64.1%), followed by vetiver (48.1%) and citronella (40.7%) oils (Table 2). When comparing the final escape proportions for the same concentration, only one significant difference was found: 5.0% DEET caused a higher escape proportion than vetiver oil at the same concentration (Fig. 2).

**Figure 3:**
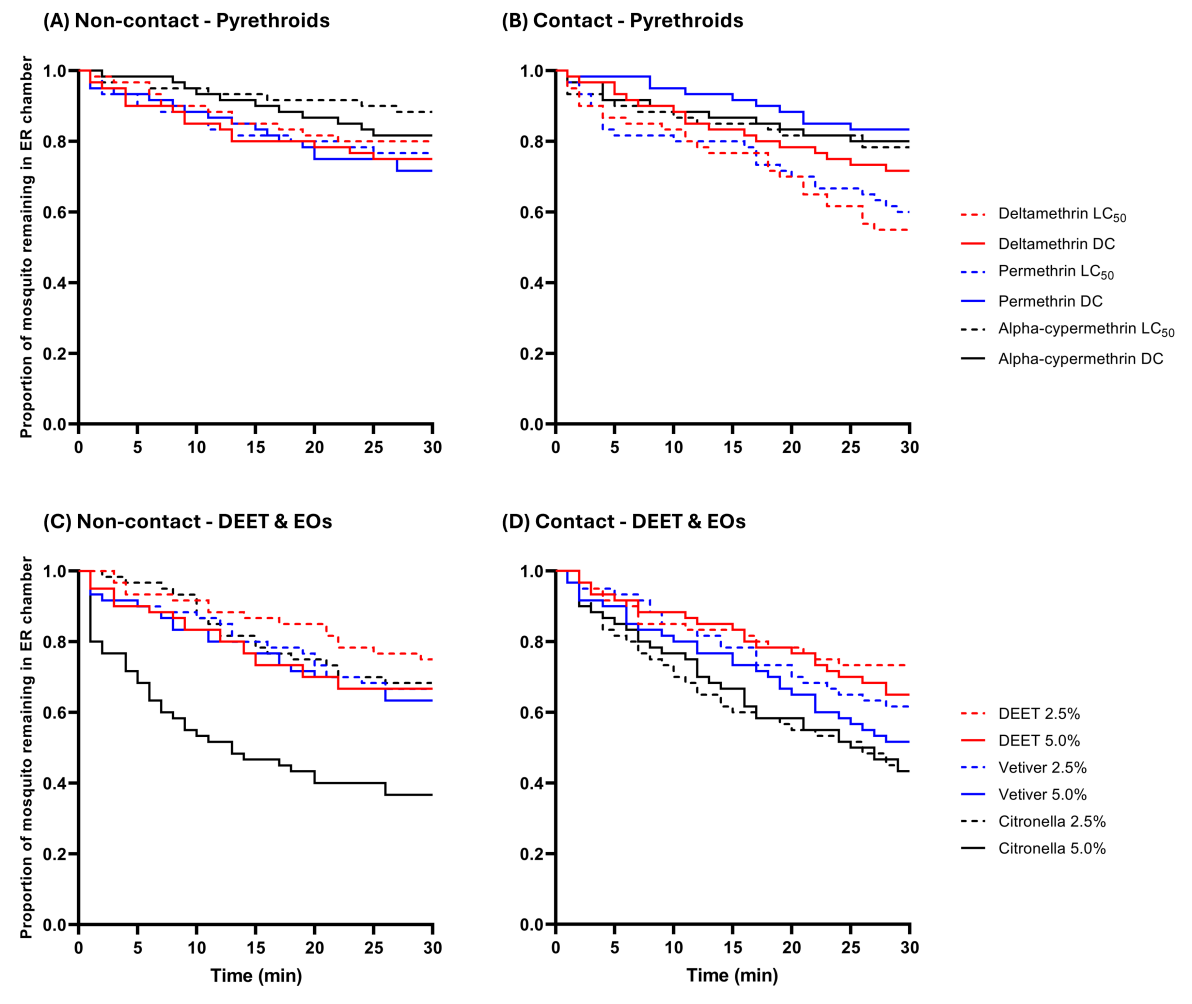
The proportion of *An. epiroticus* laboratory strain remaining in the excito-repellency chamber when exposed to synthetic (A, B) and natural (C, D) repellents. Escape responses were recorded in the treated non-contact and contact trials at 1-min intervals over a 30-min exposure period. Paired control escape responses are not shown.

**Figure 4:**
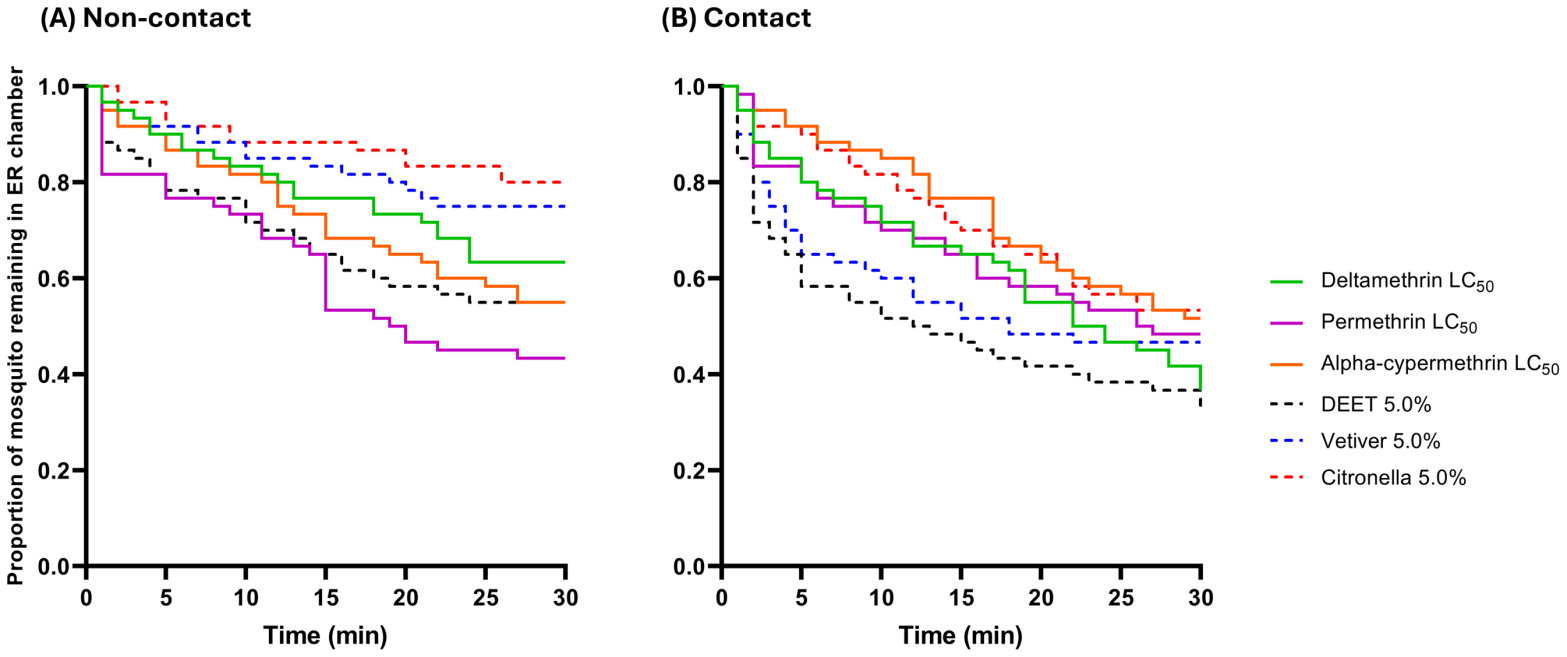
The proportion of *An. epiroticus* field strain remaining in the excito-repellency chamber when exposed to synthetic and natural repellents in the treated (A) non-contact and (B) contact trials. Escape responses were recorded at 1-min intervals over a 30-min exposure period. Paired control escape responses are not shown.

To compare the escape response patterns (time-to-escape) of *An. epiroticus*, raw escape data were used to generate survival curves, showing the mean proportion of mosquitoes remaining in the ER chambers at 1-minute intervals during the 30-minute exposure period (Figs. 3 and 4). All synthetic pyrethroids produced similar escape patterns, for the laboratory strain, regardless of concentration in both trials (Figs. 3A and 3B), although a significantly quicker escape pattern was observed at the LC_50_ of deltamethrin in the contact trials (Fig. 3B and Table 1S). In the non-contact trial, 5.0% citronella oil exhibited a more pronounced and rapid escape pattern compared to DEET and vetiver oil (Fig. 3C). Additionally, in the contact trial, *An. epiroticus* displayed a significantly higher escape pattern when exposed to both 2.5% citronella oil compared to the non-contact trial (Fig. 3D and Table 1S). Conversely, for the field population, unlike the laboratory strain, 5.0% citronella oil elicited fewer escaped mosquitoes in both trials (Fig. 4). The established LC_50_ value of permethrin produced a quicker escape pattern in the non-contact trial (Fig. 4A), whereas 5.0% DEET showed a more rapid escape pattern in the contact trial (Fig. 4B). Overall, except for permethrin and alpha-cypermethrin, all repellents produced significantly faster escape patterns in the contact trial compared to the non-contact trial (Fig. 4 and Table S2).

Multiple paired comparisons of the escape patterns of the *An. epiroticus* laboratory strain between two test concentrations was conducted for each compound using log-rank analysis (Table 3). Significant differences in escape patterns between concentration were observed for permethrin in the contact trial (LC_50_ faster than DC) and for citronella oil in the non-contact trial (5.0% faster than 2.5%). Overall, the test compounds in the present study exhibited similar excito-repellent properties between LC_50_ and diagnostic concentrations for synthetic pyrethroids, and between 2.5% and 5.0% for DEET and essential oils, in terms of both final escape proportions and escape patterns (Table 3 and Fig. 3).

Escape patterns were compared between the laboratory strain and the field strains within the same test compound, concentration, and ER trials using log-rank analysis (Table 4). Three pairwise comparisons (deltamethrin, permethrin, and alpha-cypermethrin) showed significant differences in the non-contact trial, with the field population exhibiting a faster escape pattern. In contrast, 5.0% citronella oil induced a faster escape response in the laboratory strain. In the contact trial, only two pairwise comparisons (alpha-cypermethrin and DEET) were significantly different, with the field population also showing a quicker escape pattern (Table 4). Overall, in terms of both final escape proportions and escape patterns, the findings indicate that pyrethroids and DEET exerted a stronger excito-repellency effect on the field population of *An. epiroticus*, whereas essential oils, particularly citronella oil, were more effective in repelling the laboratory strain.

**Table 3:** Statistical comparison of escape patterns and final escape proportions of *An. epiroticus* laboratory strain within repellents and ER assay configuration between concentrations.

**Repellents**	**Concentrations**	**Escape patterns** ^a^		**Final escape proportions** ^b^	
		**Non-contact**	**Contact**	**Non-contact**	**Contact**
Deltamethrin	LC_50_*vs.* DC	0.5037	0.0668	0.3714	0.0857
Permethrin	LC_50_*vs.* DC	0.5920	0.0043^*^	0.6571	0.0286^*^
Alpha-cypermethrin	LC_50_*vs.* DC	0.3213	0.8143	0.3714	0.6286
DEET	2.5 *vs.* 5.0%	0.2574	0.3993	0.3141	0.5714
Vetiver oil	2.5 *vs.* 5.0%	0.6890	0.2863	0.6286	0.4571
Citronella oil	2.5 *vs.* 5.0%	<0.0001^*^	0.8947	0.0286^*^	>0.9999

**Notes.**

a Log-rank tests were applied to Kaplan–Meier survival curves to compare differences in escape patterns (time-to-escape) over the ER assay period.

b Mann–Whitney U tests were used to compare final escape proportions at the end of the ER assay between concentrations.

* Significant difference *P* < 0.05.

% w/v means percent of weight (g) of repellent in the total volume of solution.

LC_50_; lethal concentration 50, DC; diagnostic concentration.

LC_50_ of deltamethrin, permethrin, and alpha-cypermethrin are 0.00035%, 0.01030%, and 0.00046%, respectively. DC of deltamethrin, permethrin, and alpha-cypermethrin are 0.006%, 0.349%, and 0.009%, respectively.

**Table 4:** Statistical comparison of escape patterns and final escape proportions of *An. epiroticus* within ER assay configuration, concentrations, and repellents between mosquito strainss.

**Repellents**	**Concentrations**	**Escape patterns** ^a^		**Final escape proportions** ^b^	
		**Non-contact**	**Contact**	**Non-contact**	**Contact**
Deltamethrin	LC_50_	0.0498^*^	0.0557	0.1143	0.0286^*^
Permethrin	LC_50_	0.0003^*^	0.1634	0.0857	0.5143
Alpha-cypermethrin	LC_50_	<0.0001^*^	0.0043^*^	0.0286^*^	0.0286^*^
DEET	5.0%	0.1599	<0.0001^*^	0.2857	0.0571
Vetiver oil	5.0%	0.1881	0.2255	0.1143	0.7429
Citronella oil	5.0%	<0.0001^*^	0.2996	0.0286^*^	0.4286

**Notes.**

a Log-rank tests were applied to Kaplan–Meier survival curves to compare differences in escape patterns (time-to-escape) over the ER assay period.

b Mann–Whitney U tests were used to compare final escape proportions at the end of the ER assay between concentrations.

* Significant difference *P* < 0.05.

% w/v means percent of weight (g) of repellent in the total volume of solution.

LC_50_ of deltamethrin, permethrin, and alpha-cypermethrin are 0.00035%, 0.01030%, and 0.00046%, respectively.

In the present study, knockdown and mortality of *An. epiroticus* at 30 min and 24 h post-exposure, respectively, were observed in some test runs, particularly in the contact trial (Tables 3S and 4S). No knockdown or mortality was observed in the group of mosquitoes that escaped the treatment chamber in both trials, except for the 5.0% DEET in the contact trial (Table 3S). However, mortality was observed in the non-escaped group for almost all test compounds, with the highest mortality rates in the diagnostic concentrations of the pyrethroids, ranging from 31.5% to 35.3% in the contact trial. In contrast, the highest mortality rate in the non-contact trial was observed with 5.0% citronella oil (Table 3S). For the field strain, knockdown was observed only in the non-escaped group of the contact trial with 5.0% vetiver oil. Mortality rates ranging from 21.1% to 66.0% were also observed exclusively in the non-escaped group across all test compounds in the contact trial (Table 4S).

## Discussion

This study demonstrates differential behavioral responses of *Anopheles epiroticus* laboratory and field strains to synthetic and natural excito-repellent agents. Strain-specific variations were observed, with implications for interpreting ER outcomes for malaria vector control strategies in coastal areas.

The synthetic pyrethroids tested in this study, particularly deltamethrin and permethrin, demonstrated substantial excito-repellent properties against both laboratory and field strains of *An. epiroticus*. In terms of both final escape proportions and escape patterns, deltamethrin at LC_50_ concentration elicited the highest escape response (42.11%) in the laboratory strain contact trial, while producing an even stronger response (60.00%) in the field strains. This enhanced efficacy in field populations aligns with previous studies demonstrating the robust contact irritancy of deltamethrin against various *Anopheles* species (Chareonviriyaphap et al., 2013; Kongmee et al., 2012). The observed differences between laboratory and field strains may reflect adaptive behavioral responses or physiological variations resulting from continuous exposure to environmental stressors and potential insecticide pressure in natural settings. Permethrin showed similar patterns, with notable excito-repellent activity in both populations, though with somewhat lower efficacy compared to deltamethrin. This finding is consistent with previous research indicating that deltamethrin generally exhibits stronger irritant properties than permethrin against *Anopheles* vectors (Chareonviriyaphap, Prabaripai & Bangs, 2004). Interestingly, alpha-cypermethrin demonstrated relatively weaker excito-repellent effects across both populations and trial types, suggesting potential differences in the molecular mechanisms underlying behavioral responses to different pyrethroid compounds.

Among natural repellents, citronella oil emerged as the most effective agent, particularly at 5.0% concentration, where it elicited escape responses of 59.26% and 52.73% in non-contact and contact trials, respectively, for the laboratory strain. This superior performance of citronella oil compared to other natural repellents is consistent with numerous studies demonstrating its effectiveness against various mosquito species (Asadollahi et al., 2019; Kongkaew et al., 2011; Luker et al., 2023; Nararak et al., 2016; Sathantriphop et al., 2014). The high efficacy of citronella oil can be attributed to its complex mixture of bioactive compounds, including citronellal, geraniol, and citronellol, which are known to interfere with mosquito olfactory receptors and trigger avoidance behaviors. For instance, citronellal has been shown to directly activate TRPA1 channels in mosquitoes such as *Anopheles gambiae*, triggering a neurophysiological response that leads to repulsion behavior (Kang et al., 2011). Geraniol and citronellol have demonstrated inhibitory effects on mosquito OBPs and ORs involved in host odor detection, particularly those tuned to lactic acid and CO_2_ cues (Ke et al., 2025; Wu et al., 2020).

Different response patterns were observed between laboratory and field strains. Field strains showed enhanced responsiveness to synthetic compounds, particularly in contact trials, while laboratory strains demonstrated greater sensitivity to natural repellents, especially citronella oil. This strains-specific variation suggests that field strains may have developed behavioral adaptations that affect their response to different classes of repellent compounds. The enhanced response observed in the field strain for some compounds (including pyrethroids and DEET) may reflect differences in physiological state, environmental history, or prior insecticide exposure. However, because we did not conduct resistance profiling or document prior exposure histories, these explanations should be considered hypotheses rather than confirmed drivers of the observed behavioral differences. Similar population-specific variations in behavioral responses to insecticides in other *Anopheles* species, have been attributed to the development of behavioral resistance mechanisms (Chareonviriyaphap, 2012; Mongkalangoon et al., 2009). Conversely, the stronger response of laboratory strains to natural repellents might indicate a lack of prior exposure to these compounds or retention of natural behavioral responses that may be modified in field populations through environmental conditioning.

The distinction between contact irritancy and non-contact (spatial) repellency helps clarify how different compounds drive mosquito escape behavior in the ER assay. Pyrethroids primarily act on the mosquito nervous system through neuroexcitation and knockdown, classically linked to their action on voltage-gated sodium channels; therefore, both contact and non-contact avoidance can be expressed as sublethal behavioral outcomes depending on exposure route (Andreazza et al., 2021). In our study, pyrethroids produced measurable escape responses in both contact and non-contact trials, which is consistent with a combined contribution of direct tarsal stimulation and low-level airborne exposure within the test chamber, without implying that high volatility is required. Notably, deltamethrin at the LC_5__0_ concentration produced higher escape in contact than non-contact trials in both the laboratory strain (contact: 42.11% *vs* non-contact: 12.73%) and the field strain (contact: 60.00% *vs* non-contact: 26.93%), indicating that direct contact can enhance excito-repellency for specific compound–population combinations (Mongkalangoon et al., 2009; Sathantriphop et al., 2014). Because deltamethrin, permethrin and cypermethrin have very low vapor pressures and are generally regarded as practically non-volatile, any non-contact responses observed in the ER chamber are most plausibly driven by trace airborne exposure (*e.g.*, minimal vapor/aerosol fractions) rather than high volatility (WHO, 1990). In comparison, DEET is primarily regarded as a behavioral repellent acting largely through olfactory pathways—by modulating odorant receptor signaling and/or reducing the effective delivery of host odors to olfactory receptor neurons—rather than by eliciting VGSC-mediated neurotoxicity typical of pyrethroids (DeGennaro et al., 2013). Likewise, citronella oil, a mixture rich in volatile monoterpenoids (*e.g.*, citronellal, citronellol, geraniol), is expected to produce predominantly non-contact (spatial) effects driven by volatile-mediated sensory detection, with additional contact/gustatory contributions reported for citronellal *via* TRPA1-related pathways in insects (Wu et al., 2020). Collectively, these mechanistic considerations support interpreting ER outcomes as integrated behavioral responses shaped by both molecular targets and exposure dynamics. Importantly, most contact–non-contact comparisons did not differ significantly, as indicated by shared significance letters in Tables 1–2; significant divergence was detected only for specific combinations (*e.g.*, deltamethrin at LC_5__0_ in both strains; citronella 2.5% in the laboratory strain; and vetiver 5% in the field population). Therefore, we interpret contact–non-contact contrasts as compound- and population-dependent trends rather than a consistent general pattern across all repellents.

Several limitations of this study should be acknowledged. First, the experiments were conducted under controlled laboratory conditions, which may not fully capture the complex environmental factors that influence mosquito behavior in natural settings. Second, because the number of F_1_ mosquitoes available from the field collection was limited, we were unable to perform susceptibility testing of the study field strain to pyrethroids. Although *An. epiroticus* s.l. collected on the same island approximately 3.5 km from our collection site previously showed complete (100%) susceptibility to deltamethrin, permethrin, and alpha-cypermethrin (Ritthison et al., 2014b), the susceptibility status and exposure history of the present field strain remain unknown and may not be comparable to the laboratory strain. Third, the field strain originated from a single geographic location and may not represent the full range of behavioral variation among *An. epiroticus* strains across coastal regions of Thailand. More broadly, our findings should be interpreted in light of the scope of the excito-repellency (ER) assay: the ER system quantifies an integrated behavioral outcome (escape/avoidance) under defined chamber conditions and does not, by itself, establish causal mechanisms, prior exposure histories, or population-level selection processes. Accordingly, population-specific explanations (*e.g.*, behavioral modulation, insecticide history, or selection pressure) are presented as plausible hypotheses rather than confirmed drivers because resistance profiling (*e.g.*, susceptibility assays, *kdr* genotyping, metabolic enzyme assays) and independent mechanistic/behavioral assays were not conducted. In addition, while log-rank tests can detect statistically significant differences in escape-time distributions, statistical significance does not necessarily equate to biological importance; therefore, we interpret time-to-escape patterns alongside effect-size metrics, including final corrected escape proportions (Tables 1–2) and the magnitude of divergence between conditions, rather than relying on *P*-values alone. Finally, mortality or incapacitation during the observation period can reduce the number of mosquitoes remaining “at risk” of escaping and may influence apparent escape trajectories, particularly under highly toxic treatments; thus, ER outcomes should be viewed as behavioral responses potentially shaped by both avoidance and treatment-related impairment. Taken together, we limit causal interpretations to what is directly supported by ER assay data and highlight resistance profiling and additional behavioral/mechanistic assays as priorities for future work.

Future research should investigate the long-term effectiveness of these compounds under field conditions, including their residual activity and potential for resistance development. Studies examining the synergistic effects of combining synthetic and natural repellents could provide insights into optimized formulations for enhanced protection. Additionally, investigation of the molecular mechanisms underlying the observed population-specific behavioral differences could contribute to the development of more targeted and effective vector control strategies.

## Conclusions

This study demonstrates clear evidence of differential behavioral responses between laboratory and field strains of *An. epiroticus* to synthetic and natural excito-repellent agents. Pyrethroids, particularly deltamethrin and permethrin, along with DEET, showed enhanced efficacy against field populations as contact irritants, while natural repellents like citronella oil were more effective against laboratory strains as non-contact repellents. These findings highlight the importance of considering population-specific variations in vector control planning and suggest that integrated approaches combining synthetic and natural repellents may provide optimal protection against this important malaria vector in coastal Thailand.

## Supplemental Information

10.7717/peerj.21237/supp-1 Supplemental Information 1 Statistical comparison of escape patterns and final escape proportions of *An. epiroticus* laboratory strain within repellents and concentrations between ER assay configurations of treatment group (A) Log-rank tests were applied to Kaplan–Meier survival curves to compare differences in escape patterns (time-to-escape) over the ER assay period. (B)) Mann–Whitney U tests were used to compare final escape proportions at the end of the ER assay between concentrations. *Significant difference *P* < 0.05. % w/v means percent of weight (g) of repellent in the total volume of solution. LC_50_; lethal concentration 50, DC; diagnostic concentration. LC_50_ of deltamethrin, permethrin, and alpha-cypermethrin are 0.00035%, 0.01030%, and 0.00046%, respectively. DC of deltamethrin, permethrin, and alpha-cypermethrin are 0.006%, 0.349%, and 0.009%, respectively.

10.7717/peerj.21237/supp-2 Supplemental Information 2 Statistical comparison of escape patterns and final escape proportions of *An. epiroticus* field population within repellents and concentrations between ER assay configurations of treatment group (A) Log-rank tests were applied to Kaplan–Meier survival curves to compare differences in escape patterns (time-to-escape) over the ER assay period. (B) Mann–Whitney U tests were used to compare final escape proportions at the end of the ER assay between concentrations. *Significant difference *P* < 0.05. % w/v means percent of weight (g) of repellent in the total volume of solution. LC_50_; lethal concentration 50, DC; diagnostic concentration. LC_50_ of deltamethrin, permethrin, and alpha-cypermethrin are 0.00035%, 0.01030%, and 0.00046%, respectively. DC of deltamethrin, permethrin, and alpha-cypermethrin are 0.006%, 0.349%, and 0.009%, respectively.

10.7717/peerj.21237/supp-3 Supplemental Information 3 Mean percent knockdown (KD) and mortality of female *An. epiroticus* laboratory strain at 30 min and 24 hr post-exposure, respectively, to various synthetic and natural repellents using treatment chambers of ER assay system No knockdown and mortality were found in paired controls. (A) Percentages calculated within escaped and non-escaped groups (not total per replicate). (B) Mean number knocked down or dead per test run (n = 4). % w/v means percent of weight (g) of repellent in the total volume of solution. LC_50_; lethal concentration 50, DC; diagnostic concentration. LC_50_ of deltamethrin, permethrin, and alpha-cypermethrin are 0.00035%, 0.01030%, and 0.00046%, respectively. DC of deltamethrin, permethrin, and alpha-cypermethrin are 0.006%, 0.349%, and 0.009%, respectively.

10.7717/peerj.21237/supp-4 Supplemental Information 4 Mean percent knockdown (KD) and mortality of female *An. epiroticus* field population at 30 min and 24 hr post-exposure, respectively, to various synthetic and natural repellents using treatment chambers of ER assay system No knockdown and mortality were found in paired controls. (A) Percentages calculated within escaped and non-escaped groups (not total per replicate). (B) Mean number knocked down or dead per test run (n = 4). % w/v means percent of weight (g) of repellent in the total volume of solution. LC_50_; lethal concentration 50, DC; diagnostic concentration. LC_50_ of deltamethrin, permethrin, and alpha-cypermethrin are 0.00035%, 0.01030%, and 0.00046%, respectively. DC of deltamethrin, permethrin, and alpha-cypermethrin are 0.006%, 0.349%, and 0.009%, respectively.

10.7717/peerj.21237/supp-5 Supplemental Information 5 Decaying of the raw data for *Anopheles epiroticus* (Laboratory strain)

10.7717/peerj.21237/supp-6 Supplemental Information 6 Decaying the raw data of* Anopheles epiroticus* (Field strain)

10.7717/peerj.21237/supp-7 Supplemental Information 7 Using the excito-repellency assay, synthetic agents (deltamethrin, permethrin, DEET) elicited predominant contact irritancy in field An. epiroticus, while citronella oil demonstrated superior spatial repellency in the laboratory strain

## Additional Information and Declarations

### Field Study Permissions

The following information was supplied relating to field study approvals (*i.e.*, approving body and any reference numbers): The Field experiment were approved by Kasetsart University’s Institutional Animal Care and Use Committee (Reference No. ACKU68-ETC-009).

### Data Availability

The following information was supplied regarding data availability: The raw escape data are available in the Supplemental Files.
