# Supplementary material for: Do natural or synthetic excito-repellents work better? A study on coastal malaria vector Anopheles epiroticus in Ko Chang, Thailand
Source: PeerJ. 2026 May 15;14:e21237. doi: 10.7717/peerj.21237 (PMC13182721; doi:10.7717/peerj.21237)
Supplement: Supplemental Information 1 — (A) Log-rank tests were applied to Kaplan–Meier survival curves to compare differences in escape patterns (time-to-escape) over the ER assay period. (B)) Mann–Whitney U tests were used to compare final escape proportions at the end of the ER assay between concentrations. *Significant difference P < 0.05. % w/v means percent of weight (g) of repellent in the total volume of solution. LC50; lethal concentration 50, DC; diagnostic concentration. LC50 of deltamethrin, permethrin, and alpha-cypermethrin are 0.00035%, 0.01030%, and 0.00046%, respectively. DC of deltamethrin, permethrin, and alpha-cypermethrin are 0.006%, 0.349%, and 0.009%, respectively. [file peerj-14-21237-s001.docx]

**Table 1S** Statistical comparison of escape patterns and final escape proportions of *An. epiroticus* laboratory strain within repellents and concentrations between ER assay configurations of treatment group.

| **Repellents** | **Concentrations** | **Escape patterns^a^** | **Final escape proportions^b^** |
| --- | --- | --- | --- |
| Deltamethrin | LC_50_ | 0.0053* | 0.0286* |
|  | DC | 0.7523 | >0.9999 |
| Permethrin | LC_50_ | 0.0630 | 0.1714 |
|  | DC | 0.1162 | 0.2857 |
| Alpha-cypermethrin | LC_50_ | 0.1381 | 0.1143 |
|  | DC | 0.7627 | 0.9714 |
| DEET | 2.5% | 0.7527 | >0.9999 |
|  | 5.0% | 0.9888 | >0.9999 |
| Vetiver oil | 2.5% | 0.6113 | 0.8286 |
|  | 5.0% | 0.2574 | 0.2571 |
| Citronella oil | 2.5% | 0.0042* | 0.0286* |
|  | 5.0% | 0.1542 | 0.6286 |

^a^ Log-rank tests were applied to Kaplan–Meier survival curves to compare differences in escape patterns (time-to-escape) over the ER assay period.

^b^ Mann–Whitney U tests were used to compare final escape proportions at the end of the ER assay between concentrations.

*Significant difference P < 0.05.

% w/v means percent of weight (g) of repellent in the total volume of solution.

LC_50_; lethal concentration 50, DC; diagnostic concentration.

LC_50_ of deltamethrin, permethrin, and alpha-cypermethrin are 0.00035%, 0.01030%, and 0.00046%, respectively. DC of deltamethrin, permethrin, and alpha-cypermethrin are 0.006%, 0.349%, and 0.009%, respectively.
