# Supplementary material for: Do natural or synthetic excito-repellents work better? A study on coastal malaria vector Anopheles epiroticus in Ko Chang, Thailand
Source: PeerJ. 2026 May 15;14:e21237. doi: 10.7717/peerj.21237 (PMC13182721; doi:10.7717/peerj.21237)
Supplement: Supplemental Information 3 — No knockdown and mortality were found in paired controls. (A) Percentages calculated within escaped and non-escaped groups (not total per replicate). (B) Mean number knocked down or dead per test run (n = 4). % w/v means percent of weight (g) of repellent in the total volume of solution. LC50; lethal concentration 50, DC; diagnostic concentration. LC50 of deltamethrin, permethrin, and alpha-cypermethrin are 0.00035%, 0.01030%, and 0.00046%, respectively. DC of deltamethrin, permethrin, and alpha-cypermethrin are 0.006%, 0.349%, and 0.009%, respectively. [file peerj-14-21237-s003.docx]

**Table 3S** Mean percent knockdown (KD) and mortality of female *An. epiroticus* laboratory strain at 30 min and 24 hr post-exposure, respectively, to various synthetic and natural repellents using treatment chambers of ER assay system.

| **Repellents** | **ER assay** | **%KD at 30 min^a^ (^b^)** | | **% Mortality at 24 hr^a^ (^b^)** | |
| --- | --- | --- | --- | --- | --- |
|  |  | **NEsc.** | **Esc.** | **NEsc.** | **Esc.** |
| Deltamethrin (LC_50_) | Non-contact |  |  |  |  |
|  | Contact |  |  | 5.56 (0.5) |  |
| Deltamethrin (DC) | Non-contact |  |  | 4.36 (0.5) |  |
|  | Contact | 27.53 (3.0) |  | 35.35 (3.75) |  |
| Permethrin (LC_50_) | Non-contact |  |  |  |  |
|  | Contact |  |  |  |  |
| Permethrin (DC) | Non-contact |  |  |  |  |
|  | Contact | 13.99 (1.75) |  | 31.55 (4.0) |  |
| Alpha-cypermethrin (LC_50_) | Non-contact |  |  | 3.87 (0.5) |  |
|  | Contact |  |  | 4.36 (0.5) |  |
| Alpha-cypermethrin (DC) | Non-contact |  |  |  |  |
|  | Contact | 15.35 (1.75) |  | 32.91 (4.0) |  |
| 2.5% DEET | Non-contact |  |  | 4.58 (0.5) |  |
|  | Contact |  |  | 4.01 (0.5) |  |
| 5.0% DEET | Non-contact |  |  |  |  |
|  | Contact |  |  | 12.14 (1.0) | 8.33 (0.25) |
| 2.5% Vetiver oil | Non-contact |  |  |  |  |
|  | Contact |  |  |  |  |
| 5.0% Vetiver oil | Non-contact |  |  |  |  |
|  | Contact | 10.86 (0.75) |  | 23.15 (1.75) |  |
| 2.5% Citronella oil | Non-contact |  |  |  |  |
|  | Contact |  |  |  |  |
| 5.0% Citronella oil | Non-contact |  |  | 30.71 (1.75) |  |
|  | Contact |  |  | 29.37 (1.75) |  |

No knockdown and mortality were found in paired controls.

^a^ Percentages calculated within escaped and non-escaped groups (not total per replicate).

^b^ Mean number knocked down or dead per test run (n = 4).

% w/v means percent of weight (g) of repellent in the total volume of solution.

LC_50_; lethal concentration 50, DC; diagnostic concentration.

LC_50_ of deltamethrin, permethrin, and alpha-cypermethrin are 0.00035%, 0.01030%, and 0.00046%, respectively. DC of deltamethrin, permethrin, and alpha-cypermethrin are 0.006%, 0.349%, and 0.009%, respectively.
