# Supplementary figures and images for: Do natural or synthetic excito-repellents work better? A study on coastal malaria vector Anopheles epiroticus in Ko Chang, Thailand
Source: PeerJ. 2026 May 15;14:e21237. doi: 10.7717/peerj.21237 (PMC13182721; doi:10.7717/peerj.21237)

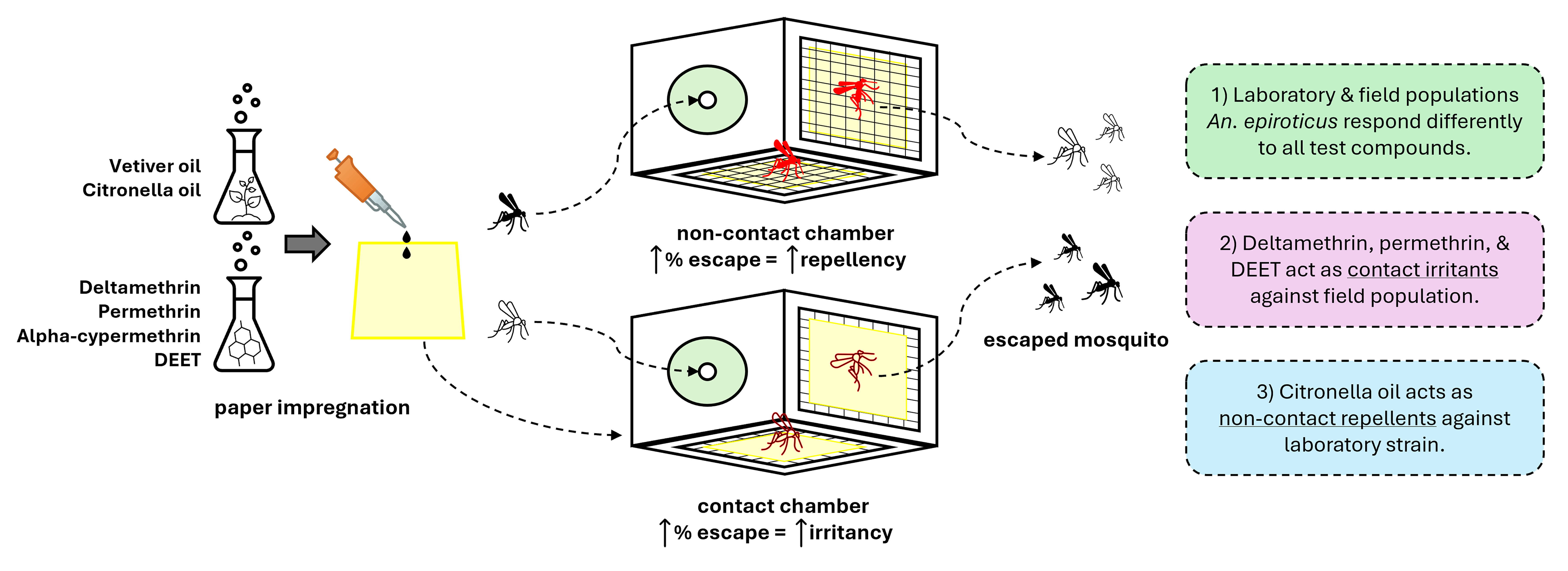

Supplement: Supplemental Information 7 [file peerj-14-21237-s007.png]
